# Iron status in the elderly

**DOI:** 10.1016/j.mad.2013.11.005

**Published:** 2014-03

**Authors:** Susan J. Fairweather-Tait, Anna A. Wawer, Rachel Gillings, Amy Jennings, Phyo K. Myint

**Affiliations:** Norwich Medical School, University of East Anglia, Norwich NR4 7TJ, UK

**Keywords:** Iron status, Elderly, Anaemia, Dietary intake, Iron stores

## Abstract

Iron deficiency anaemia is prevalent in older age, particularly after the age of 80. Serum ferritin concentrations also decline, although there is no evidence to suggest that changes in iron stores are an inevitable consequence of ageing. Chronic inflammation is a common condition in older people, making the measurement of iron status difficult, and it is likely that elevated levels of circulating hepcidin are responsible for changes in iron metabolism that result in systemic iron depletion. Other contributory factors are poor diet and some medications, such as aspirin. Anaemia in older age has undesirable health outcomes, including increased susceptibility to falling and depression. However, there are concerns about possible adverse effects of iron supplements, either in relation to pro-inflammatory effects in the gut or inappropriate tissue iron deposition. Brain iron levels are increased with age-related degenerative diseases, but it is not known if this is the cause or a consequence of the disease, and genetic factors are likely to play a role. In order to maintain body iron within the normal range a personalised approach is required, taking into account all of the factors that may affect iron metabolism and the available strategies for preventing iron deficiency or overload.

## Highlights

• Iron-deficiency anaemia is relatively common in old age.
• It is mainly caused by an inadequate diet and the presence of inflammation.
• It leads to a decline in physical performance, increased risk of falling, and depression.
• High dose iron supplements may have adverse effects.
• Iron status is difficult to measure in elderly people and there are no universally agreed ‘cut-off’ values.

## 1 Aim of this review

The objective of this narrative review was to summarise the latest information on changes in iron metabolism and status in the elderly population and consequent effects on health in order to provide the framework for studies on iron in an EC-funded project, NU-AGE (new dietary strategies addressing the specific needs of the elderly population for healthy ageing in Europe). Serum ferritin and soluble transferrin receptor will be measured in 1250 male and female volunteers between the ages of 65–79 y from five different European centres (UK, Italy, France, Netherlands, and Poland). Half of the volunteers have been randomly assigned to a one-year ‘whole diet’ intervention centred on dietary guidelines specifically tailored for elderly people with the aim of reducing age-related inflammation. The other half have been asked to maintain their usual diet for the year. Iron status is being measured at the beginning and end of the intervention to determine if a reduction in inflammatory status, resulting from the dietary changes, has an impact on iron metabolism. In addition, dietary intake data will be analysed to identify factors that explain the variance in iron status in elderly men and women, using both cross-sectional and longitudinal data.

## 2 Background

The adult human body contains 3–4 g of iron, approximately 70% of which is present in haemoglobin (Hb) in red blood cells and myoglobin in muscle. Iron is instrumental for the transport of oxygen around the body and is an essential component of many enzymes and cytochromes where it plays a role in electron transport, respiration and hormone synthesis. As a result of these multiple functions, iron is important for physical performance, immunity, cognitive development and function, thermoregulation, and thyroid metabolism. The body efficiently recycles iron from degraded red blood cells so the daily requirement to replace endogenous losses from the gastrointestinal tract, skin, hair, sweat and menstrual blood loss in women is relatively low, at about 1–1.5 mg/d.

Iron deficiency (ID) is the most common nutritional deficiency disorder in the world, defined as a lack of body iron stores, and usually caused by inadequate absorption and/or excessive iron losses. It is the result of an imbalance between iron supply and iron requirements of the erythroid bone marrow. The next stage of deficiency is iron-deficient erythropoiesis, characterised by reduced transferrin saturation. Finally, Hb concentrations fall and hypochromic, microcytic anaemia (IDA) is observed; this affects over 1 billion people worldwide (WHO, 2008).

## 3 Measurement of iron status

There are a number of biomarkers that reflect different aspects of iron metabolism and can be used singly or collectively to assess body iron status. An in-depth review of biomarkers of iron status is available on the Biomarkers of Nutrition for Development (BOND) website (www.nichd.nih.gov/global_nutrition/programs/bond).

1. Bone marrow grading is the gold standard method of assessing iron deficiency but is highly invasive so rarely used.
2. Serum iron concentration and transferrin saturation indicate the adequacy of the iron supply to developing red blood cells. Serum iron is less reliable as it is subject to diurnal rhythms and increases after the ingestion of iron-containing foods. A transferrin saturation of <15% generally indicates iron deficiency.
3. Zinc protoporphyrin (ZPP). When there is an inadequate supply of iron, zinc is incorporated into the protoporphyrin ring of the haem structure. An elevated ZPP is characteristic of iron deficient erythropoiesis.
4. Soluble serum/plasma transferrin receptor (sTfR). This binds diferric transferrin (Tf) on the cell surface. The main source of serum sTfR is bone marrow erythroid precursors (Flowers et al., 1989). When intracellular iron supply is reduced, cell surface TfR1 expression is up-regulated in order to acquire more iron, and it is down-regulated when there is sufficient iron. An elevated sTfR is a marker of tissue ID and increased bone marrow erythropoietic activity. The sTfR concentration increases in parallel with the severity of iron depletion and treatment of individuals with IDA results in a progressive fall in sTfR values (Skikne et al., 1990).
5. Serum/plasma ferritin concentration correlates closely with body iron stores, and values of <12 μg/L indicate absence of liver iron stores. However it is an acute phase protein and is elevated in people with infection or inflammation (see below). In order to identify raised ferritin values that do not accurately reflect body iron stores, C-reactive protein (CRP) or alpha-acid-glycoprotein (AGP) are determined and if these are above the normal cut-off, ferritin concentration is not used as a biomarker of iron status.
6. Body iron (Cook et al., 2003) is not a quantitative measure of iron in the body but is a sensitive index that is useful for monitoring changes in iron status, for example resulting from interventions. It is the ratio of serum transferrin receptor to serum ferritin concentration. It is a relatively new epidemiological technique for monitoring iron status in population groups susceptible to iron deficiency in which inflammation is uncommon or has been excluded by laboratory screening.
7. Hb concentration is a commonly measured biomarker but is not a specific measure of IDA as there are other causes of anaemia e.g. folate and B12 deficiency, and also the anaemia of chronic disease. It is not sensitive as the cut-off values and normal ranges vary according to sex, age, and ethnicity. Hypochromic microcytic appearance of red cells in blood film examination is used by clinician as suggestive of iron deficiency but requires a second biomarker (e.g. ferritin) to confirm the diagnosis of IDA.

## 4 Factors that affect biomarkers of status

Hb concentration has been reported to decline with advancing age, even in the absence of demonstrable disorders. In one report this was calculated to be 0.53 g/L/y in men and 0.05 g/L/y in women between the ages of 70 and 88 y (Nilsson-Ehle et al., 2000) and in another the decline was 0.1 g/L/y in men and 0.09 g/L/y in women between the ages of 70 and 80 y (Milman et al., 2008). The decline appears to increase after the age of 80, particularly in men. It has been shown that growth hormone and/or insulin-like growth factor-1 are positively and erythropoietin negatively correlated with Hb in elderly people (Nilsson-Ehle et al., 2005). Erythrocytes released from the bone marrow are less functional and partially damaged in aged individuals and as they are less able to protect themselves against stress this results in their early sequestration (Gershon and Gershon, 1988).

Differences in Hb concentration and ferritin levels have been noted between ethnicities (Patel et al., 2007). For example, anaemia is reported to be more common amongst black American compared to white American adults. However, there is evidence that the Hb distribution curve is shifted towards lower values in blacks (Perry et al., 1992) which has led to the debate about race-specific criteria for defining anaemia (Beutler and Waalen, 2006).

Cross-sectional data from the second National Health and Nutrition Examination Survey show that serum ferritin concentrations increase with age until the sixth decade of life at which time they reach a plateau (Yip, 1994). In a cross-sectional study of 441 men and women aged 60–93 y, serum ferritin concentration was reported to be positively associated with increasing age in women (*p* = 0.0223) but not in men. However, in a longitudinal study undertaken in a sub-set of 125 people there was no significant change in iron stores over the 10 y monitoring period (Garry et al., 2000), suggesting that changes in serum ferritin (and iron stores) are not an inevitable consequence of ageing.

Chronic inflammation, a common condition in older people, alters iron metabolism and haematopoiesis and can lead to anaemia (Lee, 1983), but it is difficult to determine whether or not the cause of anaemia is insufficient iron supply because indices of iron status (notably serum iron, ferritin and transferrin) are modified by the inflammatory state. It has been observed that malnutrition, not uncommon in the elderly, can exacerbate the effect of inflammation on biomarkers of iron status. Nevertheless, it is possible to differentiate between pure iron deficiency anaemia, anaemia of chronic disease, and anaemia of chronic disease with co-existing iron deficiency using the sTfR and sTfR/log serum ferritin index (Jain et al., 2010, Hanif et al., 2005). Rimon et al. (2002) undertook tests for anaemia in consecutive patients admitted to an acute geriatric ward who were older than 80 y. Bone marrow examination confirmed iron deficiency anaemia in 49 individuals but the routine laboratory tests identified only 8, whereas the transferrin receptor-ferritin index identified 35, demonstrating that this is a more sensitive and specific method for diagnosing iron deficiency anaemia in the elderly when bone marrow aspirates are not feasible. Karlsson et al. (2010) compared bone marrow iron status with various biomarkers of iron status in 50 elderly patients. The sTfR assay correctly identified 87% of iron deficient individuals; the specificity was 74%. With ferritin cut-offs of 20 μg/L for men and 7 μg/L for women this biomarker was 100% specific for iron deficiency but only 35% sensitive. When a ferritin cut-off point of 40 μg/L was used, the specificity fell to 88% but the sensitivity increased to 100%. The sTfR-ferritin index with a cut-off point of 3.0 gave a sensitivity of 100% and specificity of 43%.

Acute inflammation is another condition that affects iron metabolism. Cunietti et al. (2004) monitored changes in biomarkers through an acute inflammation episode identified by raised CRP (≥30 mg/L) in 39 older hospitalised patients (median age 79 y). All haematological indices measured, except for MCV and %transferrin saturation, were rapidly disrupted by the acute inflammation and followed differing time courses. ZPP and sTfR were not measured. The effects of a short period of inflammation (≤20 d) due to infection were similar to those observed in states of chronic inflammation.

The regular intake of aspirin, a commonly used antiplatelet agent in both primary and secondary prevention of cardiovascular diseases, is associated with lower serum ferritin. In 916 elderly people (aged 67–96 y) participating in the Framingham Heart Study, those who took > 7 aspirins/week has a significantly lower serum ferritin (*p* = 0.004), and the effect was more marked in diseased than healthy subjects (Fleming et al., 2001). The authors suggested that the effect could be related to increased occult blood loss and possibly a cytokine-mediated effect on serum ferritin in individuals with inflammation, infection or liver disease. Although samples with a CRP > 6 mg/L were excluded, it is possible that mild inflammatory states were not detected due to the low sensitivity of the CRP assay employed.

Obesity may complicate the interpretation of biomarkers of iron status. The observed hypoferraemia of obesity may be due to both true iron deficiency and inflammatory-mediated functional iron deficiency. For example, in a cross-sectional study examining the iron status of 234 obese adults compared with 172 non-obese adults attending an outpatient clinic, the obese patients had a higher prevalence of iron deficiency defined by sTfR and serum iron but not by ferritin (Yanoff et al., 2007). An increase in sTfR was reported to be associated with central obesity in men with hyperferritinemia (Freixenet et al., 2009). Tussing-Humphreys et al. (2010) measured serum hepcidin in obese premenopausal women to investigate the reason for iron depletion in obesity. They proposed that hepcidin was expressed in response to inflammation rather than changes in iron status, and concluded that the iron deficiency of obesity is true body iron deficit caused by a reduction in iron absorption rather than maldistribution of iron due to inflammation. If this is confirmed, sTfR may serve as an accurate biomarker of iron status in obese and overweight individuals.

## 5 Dietary intake

Good food sources of iron include meat and meat products which contain haem iron, especially red meat and offal and also dark poultry meat; oily fish such as tuna and sardines; cereal products such as fortified breakfast cereals; eggs; pulses; and dark green vegetables. Vegetarians avoid meat so do not consume any haem iron unless they eat fish; their main sources of iron are fortified cereals, soybeans, tofu, lentils, kidney beans, chickpeas, baked beans, dark green vegetables. Bread, potatoes and dried fruit are also a useful source of iron. In elderly people, obtaining an adequate supply of iron may be a challenge due to impaired absorption, reduced food intake associated with lower physical activity, and changes in dietary patterns that result in a more limited diet.

There are special challenges associated with collecting dietary information from elderly people, including fading memory and poor eyesight. A systematic review (Ortiz-Andrellucchi et al., 2009) selected 33 papers and categorised the studies into short-term (<7 d) or long-term (>7 d) intakes. The quality of each validation study was assessed using a scoring system, which provides a measure of accuracy of the method, and hence gave some confidence to the evaluation that was undertaken. The correlations between the different methods are summarised below, where *very good* is >0.70, *good* is 0.51–0.70, and *acceptable* is 0.30–0.50. It should be noted that the degree of correlation is dependent on the accuracy of the two methods being assessed

• FFQ vs. diet history: very good
• FFQ vs. weighed record: good
• Diet history vs. estimated record: good
• Diet history vs. weighed record: good
• FFQ vs. 24-h recall: acceptable
• Videotaped assessment vs. 24-h recall: acceptable

Declining short-term memory makes the 24-h recall method less appropriate for this age group. Diet history is a good method but extended diet history interviews should be avoided in the very old as they can take an excessively long time and concentration levels may fall. Instead these should be replaced with modified (shorter) questionnaires. Similarly, FFQs with a large number of items should be refined to reduce the number of foods.

The relationship between iron intake and iron status is complicated by variations in the efficiency of iron absorption, but a systematic review of randomised clinical trials show a positive time-dependent association between iron intake from supplemental iron and serum ferritin (Casgrain et al., 2012).

In the Framingham Heart Study, cross-sectional data of recognised modifiers of iron bioavailability were analysed in 1401 elderly men and women aged 67–95 y and positive associations with serum ferritin were observed for haem iron, supplemental iron, vitamin C and alcohol, whereas coffee intake had a negative association (Fleming et al., 1998). A study in 358 elderly Danish men and women evaluating iron status and its relationship with diet and supplement use (Milman et al., 2004) reported a positive correlation between serum ferritin and intakes of dietary iron (*p* = 0.03), meat (*p* = 0.013), alcohol (*p* < 0.001), and BMI (in men only, *p* = 0.025), and a negative correlation with tea consumption (*p* = 0.017), but no association between supplement use and iron status. Although individual dietary enhancers and inhibitors identified from single meal absorption studies (Hurrell and Egli, 2010) may have a significant impact on iron absorption and hence status, it is more important to use data on iron absorption from whole diets to predict iron status (Collings et al., 2013).

## 6 Anaemia in the elderly

In the US, it has been estimated that approximately 11% of men and 10% of women aged 65 and above are anaemic, and that these figures double at the age of 85, with prevalence rates reaching 50–60% in residential/nursing homes (Price et al., 2011). Although one of the conclusions from the Framingham Heart Study, initiated in 1948–1950, with a cohort of ∼6000 adults living in Framingham MA, was that free-living elderly white American eating a Western diet are more likely to have high iron stores, not iron deficiency (Fleming et al., 2001a), iron supplement use and high intakes of vitamin C and red meat were shown to be important determinants of iron status.

The English Longitudinal Study of Ageing, a prospective study of 3816 community-dwelling men and women (mean age 65.4 ± 9.0 y), reported that 5.2% were anaemic, and these individuals were older, less likely to drink alcohol, had a higher prevalence of morbidity, higher CRP (indicating anaemia of chronic disease) and demonstrated poorer performance on cognitive and physical function tests (Hamer and Molloy, 2009).

Large epidemiological studies report that in 30% of older people with anaemia the aetiology is unknown and the patient is diagnosed as having ‘unexplained anaemia of ageing’ (Guralnik et al., 2004). An investigation into reasons for anaemia in 190 patients aged 65 and over recruited from a haematology clinic, reported that 12% had IDA, and half of these individuals normalised Hb in response to therapeutic iron (Price et al., 2011). The ones who did not respond had malignancies (12%), renal disease (4%), or unexplained anaemia (35%). The latter had significantly higher inflammatory markers (hepcidin and ferritin, but not IL-6) than the non-anaemic controls.

## 7 Causes of iron deficiency anaemia

Anaemia in the elderly may be caused by a number of individual or combined factors, including poor diet, reduced efficiency of iron absorption, occult blood loss, medications, and chronic disease (Lopez-Contreras et al., 2010). Results from cross-sectional studies measuring the prevalence of ID and IDA in elderly people are therefore very variable.

Annibale et al. (2001) examined 668 outpatients with IDA, aged 21–94 y, and were able to identify the underlying reason for anaemia in 85% of patients. Diseases associated with bleeding were found in 37%, including colon cancer, gastric cancer, peptic ulcer, hiatus hernia with linear erosions, colonic vascular ectasia, colonic polyps and Crohn's disease. Causes not associated with bleeding were found in 51%, including atrophic gastritis, celiac disease, and *Helicobacter pylori*. The authors concluded that gastrointestinal diseases not associated with bleeding are frequently associated with IDA in patients without gastrointestinal symptoms or other potential causes of gastrointestinal bleeding. The diseases observed in older patients were hiatus hernia, gastric cancer, colon cancer, and colonic vascular ectasia.

*H. pylori* infection has been implicated as a risk factor for iron deficiency, but this may be a strain-related effect (Yokota et al., 2012). A study in 220 Australian men and women aged 65 y or older reported no difference in median serum ferritin concentrations (or Hb) between *H. pylori* infected and uninfected individuals but serum ferritin concentrations were significantly lower in infected women who took low-dose aspirin (*p* = 0.04) (Kaffes et al., 2003). The authors conclude that it may be the combination of *H. pylori* infection and use of low-dose aspirin that impact on iron stores.

Institutionalisation is another known risk factor. In 252 institutionalised elderly Spanish men and women, aged 65–96 y, 4-d weighed food records were collected and iron status measured (Lopez-Contreras et al., 2010). The prevalence of anaemia was 25.4% using a Hb cut-off of <130 g/L for men and <120 g/L for women (WHO, 2001), but only 3.6% had serum ferritin below the cut-off of 15 μg/L. There was a high prevalence of inflammation/infection as illustrated by the fact that 41% of individuals had raised CRP values (>5 mg/L), and there was a significant correlation between CRP and ferritin (*p* = 0.023) but not with Hb. Diet was not one of the principal causes of anaemia in their study, except for folate intake, but it appears that infection/inflammation was a key component. In another study in Spain (Vaquero et al., 2004) in which elderly people living in retirement homes were consuming an Atlantic-Mediterranean diet (rich in meat products, fish, vegetables, fruit, olive oil and dairy products, but poor in cereals), the prevalence of anaemia was only 6.7%, but there were no measurements of infection/inflammation.

## 8 Consequences of iron deficiency

Anaemia is associated with numerous health implications, including a decline in physical performance, cognitive impairment, increased susceptibility to falling, frailty, and mortality (reviewed by Price et al., 2011).

A study in 1156 community-dwelling Italian people aged 65 y and older showed a clear association between anaemia and disability, poorer physical performance and lower muscle strength (Penninx et al., 2004) but there is no proof of causality in cross-sectional epidemiological studies. In a 4-y prospective study in men (∼30%) and women (∼70%) aged 71 y and older (Penninx et al., 2003) various physical performance tests were undertaken at baseline and after 4 y and the individuals classified for anaemia using Hb and MCV values. The number of volunteers who undertook both sets of tests was 1146. At baseline 5.9% had anaemia (Hb < 130 g/L men, <120 g/L women), and 15% had borderline anaemia (Hb 130–140 g/L men, 120–130 g/L women). Those with anaemia were older and performed significantly worse on the baseline physical performance battery. There was a predicted fall in the physical performance score (*p* for trend 0.002), which was most marked in the anaemic group (*p* = 0.003) followed by the borderline anaemic group (*p* = 0.02). The results of this study suggest that anaemia in old age is an independent risk factor for decline in physical performance.

The association between serum iron status, cardiovascular disease and all-cause mortality was examined in 336 elderly Taiwanese men and women (aged > 65 y) living in long-term care facilities (Hsu et al., 2013). The degree of iron deficiency was defined according to serum iron, but it should be noted that serum iron is affected by inflammation, as commonly present in cardiovascular disease, and it is not a good measure of iron deficiency. Although there was a positive association between low serum iron, cardiovascular disease and all-cause mortality, causality cannot be inferred. The authors propose several mechanisms for the link between iron deficiency and cardiovascular disease. Firstly, iron deficiency is associated with cancer, renal failure and chronic inflammation, which are generally accompanied by higher mortality; an iron deficient state may reflect undiagnosed conditions. Secondly, iron deficiency may be a surrogate marker for malnutrition, for which there is a link with mortality. Thirdly, iron deficiency may promote oxidative damage, for example, as illustrated by elevated serum malonyldialdehyde production in iron deficiency anaemia (Coghetto Baccin et al., 2009).

Mørkedal et al. (2011) assessed sex-specific associations of iron status with ischaemic heart disease (IHD) mortality in a prospective study of 640,798 healthy Norwegian adults. Low iron status, particularly in the early stages of follow-up, was associated with increased risk of death from IHD, but the limited range of biomarkers of iron status (serum iron, transferrin saturation, and total iron binding capacity) were measured in non-fasting blood samples, and it is possible that low iron status was a late sign in the pathogenesis of IHD or that underlying disease influenced the results.

A smaller prospective study was undertaken in Finland in which 361 men and 394 women aged 65–99 y were followed for up to 10 y (Marniemi et al., 2005), Individuals in the highest tertile for baseline serum iron had a reduced risk of acute myocardial infarction (AMI) (RR 0.544, 95% CI 0.35, 0.88) and those in the middle tertile had a reduced risk of stroke (RR 0.474, 95% CI 0.25, 0.89). There was an increased risk of stroke in those in the highest tertile of serum transferrin (RR 1.67, 95% CI 1.07, 2.61), but no effect of blood Hb on either AMI or stroke. When the relationship with serum iron was analysed taking into account additional coronary heart disease risk factors the RR of AMI and stroke were still significant (*p* = 0.016 and 0.047 respectively). In contrast, however, when the relationship between nutritional status and all-cause mortality was investigated in a prospective study of 405 community-dwelling Scottish men and women aged 75 y and older, no association was found between iron status and mortality (Jia et al., 2007).

Racial variation in the relationship of anaemia with mortality and mobility disability among older adults has been observed. In a group of 1018 black and 1583 white US adults, aged 71–82 y, anaemia was associated with higher mortality in white but not black people. Anaemia was significantly associated with increased risk of death and mobility disability in community-dwelling older whites, whereas older blacks were not at risk of adverse events. The authors suggest that the criteria for defining anaemia may need revising (Patel et al., 2007).

A cross-sectional study in 1875 men and women aged 65 y and older (enrolled in the 2005 Health Survey for England) was undertaken to examine the relationship between iron status (defined according to biochemical criteria) and symptoms of depression (Stewart and Hirani, 2012). Anaemia (Hb < 130 g/L in men, <120 g/L in women) was present in 10.8% of 1833 samples analysed, low ferritin (<45 μg/L) in 21.6% of 1851 samples analysed, moderately raised sTfR (>2.3 g/L) was present in 7.6% of the 1875 samples analysed. Depressive symptoms were significantly higher in participants with anaemia, low ferritin and high sTfR, after adjustment for age, sex, social class, multivitamin intake, smoking status and BMI, but the association was reduced substantially after further adjustment for physical health status (chronic illness). There was a significant association between higher number of depressive symptoms and lower Hb and higher sTfR but not with ferritin, which suggests that the association with anaemia is accounted for by physical health status and thus may primarily reflect anaemia of chronic disease. Onder et al. (2005) also found an association between anaemia and depression in 986 older adults from the InCHIANTI study, a prospective population-based study of community-dwelling men and women (mean age 75 y). Anaemia was present in 15% of the participants with depression and in 8% of the participants without depression (*p* < 0.001), and the risk of anaemia progressively and significantly increased with severity of depression.

## 9 Adverse effects of iron

Concern has been expressed about potential adverse effects of moderately elevated iron stores in middle-aged and older people in that they may be associated with increased risk of several chronic diseases, such as heart disease, cancer and type 2 diabetes mellitus. Increased tissue iron stores have been implicated in risk of diabetes mellitus and decrease in insulin sensitivity, but the mechanism is uncertain (Sung et al., 2012). Iron has well-described pro-oxidant effects in vitro, and may have pro-inflammatory effects. For example, Sung et al. (2012) reported that in 12,033 Korean men (mean age 41.2 y in the group with no coronary artery calcium and 47.7 y in those with coronary artery calcium) ferritin was independently associated with the presence of coronary artery calcium, a biomarker of preclinical atherosclerosis. Those in the highest quartile for ferritin (>257 μg/L) had a higher score for coronary artery calcium than the lowest quartile (<128 μg/L). However, the relationship with transferrin saturation was weaker, suggesting that there may be another contributory factor, unrelated to high iron status, e.g. inflammation or metabolic stress.

The explanation for the presence of high iron stores was sought in a study of 614 elderly Americans participating in the Framingham Heart Study. After excluding individuals with raised CRP (>6 mg/L, *n* = 45), infection (diagnosed from white blood cell count, *n* = 40) and other confounding factors (*n* = 69), 11.4% of the remaining 460 participants were reported to have elevated iron stores (serum ferritin > 300 μg/L in men, and >200 μg/L in women). Dietary factors associated with high iron stores included use of iron supplements (≥30 mg/d), >3 servings of fruit or fruit juice/d, and >4 servings of red meat/week. High intakes of wholegrain (>7 servings/week) were inversely associated with risk of having high iron stores (Fleming et al., 2002).

Three prospective studies reported an association between moderately elevated serum ferritin concentration (>200 μg/L) and risk of acute myocardial infarction (Salonen et al., 1992, Tuomainen et al., 1998, Klipstein-Grobusch et al., 1999), but the association was not observed in other prospective studies (Mänttäri et al., 1994, Frey and Krider, 1994, Magnusson et al., 1994). Furthermore, in a matched, nested case–control study (252 cases and 499 controls) drawn from the Copenhagen City Heart Study and the Copenhagen General Population Study, markers of iron overload (high serum iron and transferrin saturation) were associated with *reduced* risk of a near-term (4 y onset) myocardial infarction; conversely there was an association between low serum iron and transferrin saturation and increased risk of a near-term myocardial infarction in this apparently healthy population (Nordestgaard et al., 2010).

A few early epidemiological studies indicated a weak positive association between very high body iron levels and risk of cancer e.g. colorectal cancer (Knekt et al., 1994), possibly related to free radical tissue damage caused by iron released from degradation of tissue ferritin. The UK Scientific Advisory Committee on Nutrition has concluded that there are insufficient data to reach a clear conclusion about high iron levels and risk of cancer (Scientific Advisory Committee on Nutrition, 2010). There may, however, be an interaction between high body iron levels and lipids (either high VLDL-cholesterol or low HDL-cholesterol) that promotes oxidative stress (Mainous et al., 2005). There is emerging evidence to suggest that luminal iron in the gut may be pro-inflammatory, and iron supplements given to treat iron deficiency anaemia, common in irritable bowel disease and other gut disorders associated with faecal blood loss, may exacerbate the inflammatory processes by stimulating the production of reactive oxygen species and inflammatory cytokines (Weiss, 2011). The form of iron appears to be important, for example chelated forms of iron are better tolerated than ferrous sulphate, presumably because it generates a lower concentration of soluble iron in the lumen (Liquori, 1993).

In healthy individuals brain iron levels increase with age (Bartzokis et al., 1994) and abnormally high brain iron levels are observed in age-related degenerative diseases. For example, in Alzheimer's Disease hippocampal iron is increased beyond levels of non-demented controls (Pankhurst et al., 2008). The hippocampus is the key region in memory function that is severely affected in ageing and dementing disorders. Although there is no proof of a causal relationship, the fact that elevated levels have been observed in preclinical disease (Smith et al., 2010) has generated the hypothesis that an accelerated trajectory of brain iron accumulation, and the associated oxidative damage, may occur during the transition from healthy ageing to dementia. Bartzokis et al. (2011) measured hippocampal iron in healthy older people (aged 55–76 y) of mixed racial origin and found that in men, but not women, there was a significant decrease in memory function with increase hippocampal ferritin iron (*p* = 0.003), and independent of gender, worse verbal working memory performance was associated with higher basal ganglia iron (*p* = 0.005) in individuals without the H63D and TfC2 gene variants. The authors suggest a combination of genetic and MTI biomarkers may be useful for identifying high risk groups for primary prevention clinical trials.

## 10 Iron requirements in the elderly

Although physiological iron requirements do not differ between adult and elderly men and post-menopausal and elderly women there is growing evidence that iron metabolism is affected by the ageing process. Chronic low-grade inflammation leads to less efficient absorption through hepcidin regulation. Identifying iron deficiency becomes more of a problem because of age-related changes in Hb, effects of medication prescribed for age-related disorders and diseases, and increased ferritin concentrations associated with inflammatory states. The sTfR-ferritin index appears to be the most useful method for detecting iron deficiency in older people. There is sufficient evidence linking iron deficiency with adverse health effects to justify correcting it through diet or iron therapy, but at the same time it is important to ensure that the risk of high body iron stores is not increased as this may have detrimental effects on the brain.

## 11 Future work

One of the most challenging problems with studies of iron metabolism in older people is measuring iron status in the presence of inflammatory conditions, such as obesity and age-related chronic and degenerative diseases. Therefore the development of improved biomarkers of iron status must be given high priority. This would enable the relationships between anaemia and iron deficiency and chronic diseases to be characterised more accurately and causality clarified, and would also facilitate research on the potential links between iron and dementia. There is still some uncertainty about iron requirements in the elderly, confounded by effects of inflammation on iron status, which better biomarkers would help address. Finally, there is virtually no information about how ageing of the GI tract affects iron absorption, and this is urgently required for calculating average requirements for iron (using the factorial method) to derive dietary reference values and develop dietary recommendations for elderly populations.
